# Genetic moderation on the relationship between brain white matter hyperintensities and amyloid‐beta

**DOI:** 10.1002/alz70855_103613

**Published:** 2025-12-24

**Authors:** Yuen Yan Wong, Che‐Yuan Wu, Daniel K Mori‐Fegan, Shiropa Noor, Lisa Y. Xiong, Si Won Ryoo, Myuri Ruthirakuhan, Saira S. Mirza, Mario Masellis, Ekaterina Rogaeva, Yutaka Amemiya, Arun Seth, Joel Ramirez, Christopher JM Scott, Fuqiang Gao, Sean Symons, Chinthaka Chris Heyn, Benjamin Lam, Katherine Zukotynski, Aparna Bhan, Richard H. Swartz, Demetrios J. Sahlas, Maged Goubran, Julia Keith, David A. A. Bennett, Sandra E. Black, Meghan J. Chenoweth, Walter Swardfager

**Affiliations:** ^1^ University of Toronto, Toronto, ON, Canada; ^2^ Sunnybrook Research Institute, Toronto, ON, Canada; ^3^ Sunnybrook Health Sciences Centre, Toronto, ON, Canada; ^4^ Heart and Stroke Foundation Canadian Partnership for Stroke Recovery, Toronto, ON, Canada; ^5^ Tanz Centre for Research in Neurodegenerative Diseases, University of Toronto, Toronto, ON, Canada; ^6^ McMaster University, Hamilton, ON, Canada; ^7^ University of British Columbia, Vancouver, BC, Canada; ^8^ ICES, Toronto, ON, Canada; ^9^ Rush Alzheimer's Disease Center, Chicago, IL, USA; ^10^ Toronto Dementia Research Alliance, Toronto, ON, Canada; ^11^ Campbell Family Mental Health Research Institute, Centre for Addiction and Mental Health, Toronto, ON, Canada; ^12^ KITE Toronto Rehabilitation Institute, Toronto, ON, Canada

## Abstract

**Background:**

Brain white matter hyperintensities (WMH) are vascular lesions commonly observed in Alzheimer's disease (AD). WMH were previously shown to be associated with greater brain amyloid, but the molecular pathways underlying their complex relation remain unclear. Here, we aim to identify single nucleotide polymorphisms (SNP) that modify the relationship between WMH and AD amyloid biomarkers.

**Method:**

We conducted a genome‐wide interaction study in participants with AD, mild cognitive impairment, and normal cognition from the Alzheimer's Disease Neuroimaging Initiative (ADNI). WMH were measured from FLAIR MRI using an automated atlas‐based segmentation. Amyloid‐β 42 (Aβ42) in cerebrospinal fluid (CSF) were measured using immunoassays. Interactions between SNPs and WMH volumes on CSF‐Aβ42 were assessed via a linear regression model adjusting for age, sex, diagnosis, MMSE, *APOE*‐ε4 status, head‐size, and 4 genetic principal components in PLINK2. The most influential SNP was identified via Sum of Single Effects (SuSiE) regression. Significant SNP‐WMH interactions were validated in participants from the UK Biobank (UKB) with available plasma‐Aβ42 data quantified by liquid chromatography‐mass spectrometry. SNP‐WMH interactions in relation to amyloid pathology (diffuse and neuritic plaque burden) was investigated in the Religious Orders Study/Rush Memory and Aging Project (ROSMAP).

**Result:**

A 28‐variant intergenic locus on chromosome 18 (top SNP: rs72899960 T>A, *p* = 5.66x10^‐9^, MAF=11.1%, Imputation R^2^>0.99%, *n* = 863) interacted with WMH to predict Aβ42 (nearest gene: U7 small nuclear RNA (snRNA) *XR_007066478.1* [‐111KB]). The effect of this SNP was also significant in the dominant and recessive genetic models (relative to the minor A‐allele). This SNP‐WMH interaction was replicated in UKB (*n* = 645) in both additive (B=0.91, *p* = 0.017) and dominant models (B=0.96, *p* = 0.024). In ROSMAP (*n* = 195), significant SNP‐WMH interaction was observed on diffuse plaque burden in the additive (B=‐0.46, *p* = 0.049) and dominant (B=‐0.51, *p* = 0.046) models. The minor A‐allele exhibited a protective effect by being associated with higher circulating Aβ42 (in ADNI and UKB) and lower diffuse plaque burden (in ROSMAP) in individuals with greater WMH.

**Conclusion:**

Genomic variants moderated the relationship between WMH and amyloid biomarkers, suggesting a novel regulatory role for snRNA. This genome‐wide interaction study highlights the potential contributions of snRNA and related pathways to the relationship between vascular disease and amyloid biomarkers in AD.

